# Feasibility and acceptability of PDConnect, a multi-component intervention to support physical activity in people with Parkinson's disease: A mixed methods study

**DOI:** 10.1177/1877718X251324415

**Published:** 2025-03-28

**Authors:** Julie Jones, Lyndsay Alexander, Elizabeth Hancock, Kay Cooper

**Affiliations:** 1 School of Health Sciences, Robert Gordon University, Aberdeen, UK

**Keywords:** Parkinson's disease, physical activity, behavior change, self-management

## Abstract

**Background:**

Physical activity (PA) is beneficial for people with Parkinson's (PwP); however, many are classed as sedentary. PDConnect is an online multicomponent intervention combining 1:1 physiotherapy and group-based PA combined with education, behavior change and self-management strategies, promoting PA and self-management among PwP.

**Objective:**

To assess feasibility and acceptability of PDConnect.

**Methods:**

Mixed methods study involving 31 PwP randomly allocated to: (i) usual care: physiotherapy once a week for six weeks, and (ii) PDConnect: physiotherapy once a week for six weeks, followed by 12 weekly sessions of group-based PA, followed by three monthly Teams calls to support engagement. Outcomes included intervention feasibility and acceptability (primary) assessed via survey and interviews; PA, motor, non-motor symptoms, and health and well-being (secondary) assessed at baseline, and at six, 18, and 30 weeks. Fidelity was accessed by post hoc video analysis.

**Results:**

Online delivery of PDConnect was feasible and safe. Participant retention was 74%. Response rate of self-reported measures was 97%. 95% of participants returned completed activity diaries. Attendance was high, with all participants recommending PDConnect. PDConnect participants reported improved flexibility, muscle strength, and endurance as well as increased PA confidence, PA levels and knowledge of Parkinson's disease. Half of PDConnect participants reported that they were much improved compared to 10% of usual care participants. Small to large effect sizes in PA (d = 0.03) and UPDRS (d = 0.96) ES) were reported, which warrant further exploration in an appropriately powered study.

**Conclusions:**

PDConnect is feasible and acceptable among PwP. A future large-scale trial is required to determine the effectiveness of PDConnect.

## Plain language summary

Physical activity (PA) has been shown to beneficial for people with Parkinson's (PwP). Thus far, research has focused on examining a single form of PA with very few studies looking at how best to support PwP to get active and stay active. This new research examined whether an intervention called PDConnect would be feasible and acceptable for PwP. PDConnect is online intervention combining six weeks of 1:1 Parkinson's specialist physiotherapy, followed by 12 weekly sessions of group-based PA which included education designed to promote PA levels and self-management skills for PwP. The researchers were also interested in collecting preliminary data about whether participation in PDConnect would impact Parkinson's symptoms and their health and well-being. Thirty-one PwP participated in the study and were divided into two groups: one group received PDConnect and the other received 6 sessions of Physiotherapy. PDConnect was found to be feasible and acceptable by participants. No adverse events occurred. PDConnect participants reported improved flexibility, muscle strength, and endurance as well as increased PA confidence, PA levels and knowledge of Parkinson's. Half the PDConnect participants also reported that they were much improved compared to 10% of usual care participants. This study has shown that PDConnect is feasible and acceptable among PwP, and now the researchers would like to plan a future larger study to explore how effective PDConnect is compared to other physical activity interventions.

## Introduction

Globally, Parkinson's disease (PD) is the second most common neurodegenerative condition, after Alzheimer's disease.^1^ The prevalence of PD is predicted to rise by 50% by 2030,^2^ highlighting the need for effective healthcare interventions to manage this condition. Despite major advances in the understanding of PD, the cure remains elusive. The management of PD is complex, owing to its progressive nature, patient heterogeneity, and symptom diversity. Management is reliant on medication; however, pharmacological management neither targets underlying pathological processes, nor limits progression of the condition.^3^ Therefore, with a growing PD population, and finite benefit of medication, the development of effective long-term health interventions is crucial.

Physical activity (PA) has been hailed as the new medicine for PD.^4^ The interest in PA has been fueled by the association between PA and reduced risk of developing PD^5^ and its potential to attenuate symptom progression.^5–8^ Systematic reviews demonstrate that PA results in improved strength, balance, gait, and physical capacity, as well as improved motor and non-motor symptoms.^9–15^ High intensity PA is hypothesized to promote neurogenesis, synaptogenesis, dopamine turnover, and to reduce neuroinflammation,^16–18^ suggesting that PA may infer a neuro-restorative or protective function.^6^ Consequently, PA is currently regarded as the most positive avenue towards disease modification.^19,20^

Despite a large body of evidence advocating PA, people with Parkinson's (PwP) have been shown to be less active than age-matched peers without PD.^21^ Typically, PwP are sedentary for 75% of the time, with PA levels declining from diagnosis.^22^ In contrast, PwP who are more active experience slower decline of PD symptoms, and experience improved health outcomes,^6,23,24^ quality of life (QoL),^25^ and lower incidence of falls and fractures.^26^ Moreover, the benefits of PA extend beyond the purely physical. Physical activity also serves as a conduit for social networking and shared experience, which are highly valued by PwP.^27^ To date, research has focused on the effectiveness of different types of PA. Comparatively little research has explored the optimal means of delivering PA, with the aim of influencing PA behavior and supporting long-term PA, which may explain why PwP are aware of the benefits of PA yet remain inactive. Therefore, there is a need for effective PA interventions that can be sustainably delivered within existing health services, which are accessible to PwP to support changes in PA behavior.

Simply instructing PwP to be active is not effective.^28^ Rather, PA needs to be delivered as a package of care that can be delivered long-term to support the development of a PA habit.^29^ Self-management programs have been proposed as the mechanism to effectively manage long-term conditions and support sustained behavior change.^30^ Evidence advocates that self-management should be a multi-component intervention encompassing strategies to support changes in behavior, skill-based training, and contextualized education.^30–32^ Combining exercise with behavior change and self-management strategies, as a combined health intervention, may therefore be more effective than exercise prescription alone. However, PwP report that access to exercise and ability to self-manage are their greatest unmet needs.^33^

Following consultation with the PD community and exercise professionals with expertise in PD, a multi-component PA intervention was developed combining 1:1 and group-based evidence-informed exercise, education, and self-management strategies delivered by staff with expertise in PD. The intervention was underpinned by the COM-B behavior change model,^34^ and incorporated behavior change techniques (BCTs)^35^ selected to support the development of PA self-efficacy, increased PA participation, and PA self-management. Following the Medical Research Council (MRC) guidelines for the assessment of complex interventions,^36^ here we report the feasibility and acceptability of the PDConnect intervention. In addition, this study sought to estimate effect sizes for PA, motor, and non-motor symptoms, QoL, and health and well-being measures to inform future research.

## Methods

### Study design

**Figure 1.**
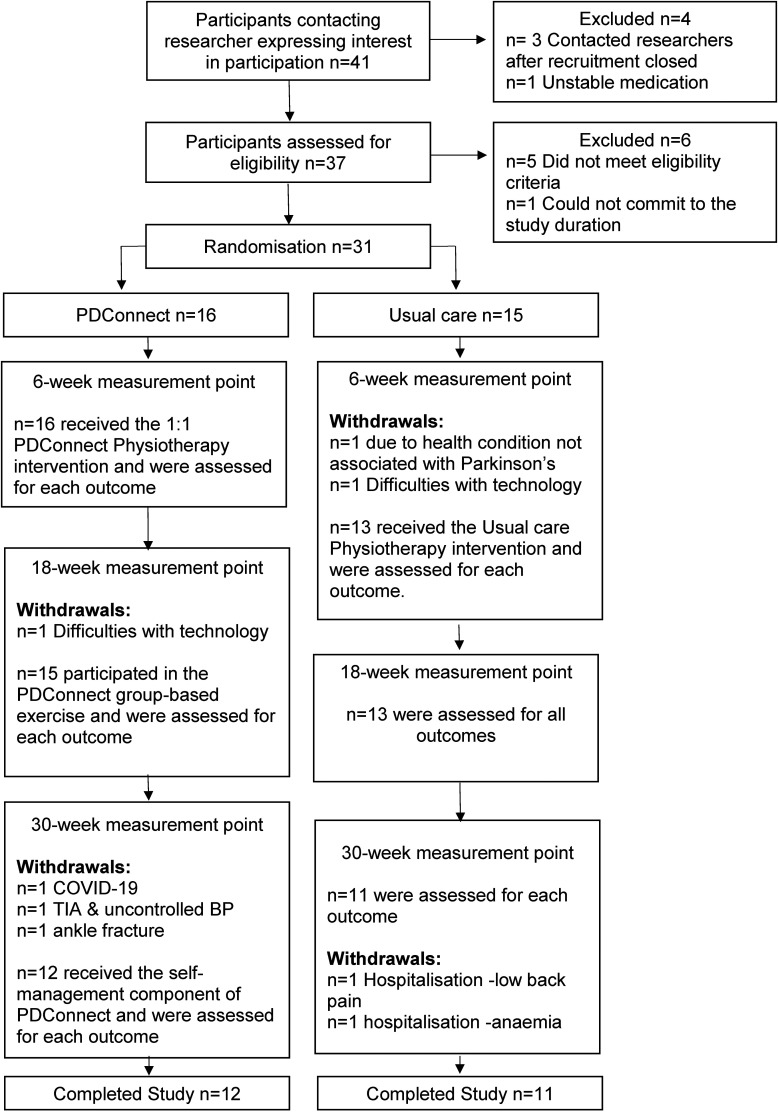
Consort flowchart: participant recruitment and retention.

A randomized controlled feasibility study adopting a parallel convergent mixed methods design was conducted between December 2020 and October 2021. Adopting this approach allowed the generation of knowledge on several aspects: feasibility, acceptability, fidelity, perceptions, adherence, and attitudes to the intervention and its processes to inform future research. Eldridge et al. (2016)^37^ define a feasibility study, as a precursor to a large-scale study, with the principal aim of establishing whether a study can be done, allowing for the exploration of processes, procedures, and intervention perceptions, to inform refinements prior to full scale evaluation.^37^ The intended outcome of this study, therefore, was to inform the development of a robust protocol and adequately powered randomized controlled trial (RCT) to evaluate the clinical and cost-effectiveness of the intervention. Acceptability was evaluated alongside feasibility to explore whether the intervention was acceptable from the perspective of those receiving or delivering it. Sekhon, Cartwright, and Francis (2017, pp8) define acceptability as “*a multifaceted construct, which is formed based on anticipated or experienced cognitive or emotional responses to an intervention*”.^38^ Adopting this definition, acceptability was explored through both quantitative and qualitative means, to inform the design, development, and implementation of a future trial. Gearing et al. (2011) defined fidelity as the degree to which an intervention is delivered as intended.^39^ Without intervention fidelity, uncertainty exists as to whether observed effects are attributable to the intervention or not.^40^ Adopting a study design incorporating feasibility, acceptability, and fidelity allows greater insight into the delivery and the experience of the health intervention. Figure 1 outlines participant flow and study processes. The study was prospectively registered (ISRCTN 11672329)**,** approved by the Liverpool Central Research Ethics Committee (20/NW/0236) and NHS Grampian Research and Development department (Ref 2020RG001E).

### Sample

Participants were invited to take part by neurologists or recruited via the Parkinson's UK Take Part Hub with no restriction on age. Participants were included if they had a confirmed diagnosis of idiopathic PD, Hoehn and Yahr stages I-III, had no medical restriction that would prevent safe exercise and were able to walk more than 100 m with or without a walking aid. Participants with co-existing neurological or musculoskeletal conditions that would restrict PA and those with uncontrolled or unpredictable on-off periods were excluded. All participants were screened using an adapted version Physical Activity Readiness Questionnaire (PAR-Q) to ensure eligibility. Adaptations included PD specific question such as presence of dyskinesia to ensure participant safety to participate in the study. All participants provided informed consent prior to taking part in the study. Participants were randomly allocated to receive usual care or PDConnect by an independent Chartered Statistician using stratified random sampling by gender and PA level to ensure balance between groups at baseline. The researcher conducting outcome assessments (JJ) was blinded to randomization. Purposeful sampling was used to recruit staff to deliver the usual care and intervention arms of the study. Physiotherapists were required to have a minimum of two-years clinical experience including neurology and geriatrics. Physiotherapists were randomly assigned to deliver either PDConnect (n = 2) or usual care (n = 2), using sealed envelopes. Two fitness instructors were recruited from the University Sport facility and had a Register of Exercise Professionals accredited (or equivalent) qualification. The two physiotherapists and fitness instructors delivering PDConnect participated in a bespoke training package involving directed individual learning, and group-based workshops prior to commencing the study, aimed at developing expertise in exercise prescription for PwP and BCTs. Total training duration was 12 h over a four-week period. The Physiotherapists delivering usual care did not receive any training.

### PDConnect intervention

PDConnect is an evidence-informed PA intervention aimed at providing PwP with a toolkit of BCTs to promote PA participation and PA self-management. It combines specialist physiotherapy, group-based PA, and self-management, with education underpinned with BCTs. The program consists of three components: i) six sessions of one-to-one sessions with a PD specialist physiotherapist; ii) 12 weekly sessions of group-based PA delivered by a fitness instructor; iii) 12 weeks of self-management, with monthly telephone contact by the same fitness instructor. All participants completed a home risk assessment and received a Microsoft Teams^®^ induction prior to commencing the study, which was delivered online due to coronavirus restrictions in place at the time.

### 1:1 physiotherapy

Each one-hour session comprised of individualized exercise prescription delivered in conjunction with education, self-management strategies, and BCTs, using a coaching style of delivery. Each session consisted of a minimum of 35 min of exercise encompassing strength, balance, aerobic, flexibility, cognitive, gait, and functional components, as per current guidelines.^41^ Exercise was progressed on an individual basis, by increasing repetitions, speed, load, or task complexity.^41,42^ One-to-one sessions were supplemented with an individualized home exercise plan (HEP). Participants were encouraged to complete their HEP for 30 min, five times a week, and record all activity within an activity diary. Development of a PA routine was promoted using an activity planner, self-completed activity diaries and self-monitoring of PA using an activity tracker. A PDConnect manual was provided to reinforce key components of the intervention and served as an educational resource. Education included PD pathophysiology, benefits of activity, types of PA, behavior change, health and safety, and tips for getting started and staying active.

### Group-based exercise

Each session included a minimum of 60 min exercise adopting a circuit-based approach. Exercise stations included mobility, strengthening, aerobic, balance, cognitive, and goal-oriented components with an emphasis on large amplitude movements and intensity of effort as recommended by current guidelines,^41,42^ and had four levels of difficulty, allowing tailoring to individual ability. Participants were provided with key teaching points, practical demonstration, and proposed benefits of each exercise prior to commencing. Exercise was followed by 30 min of group-based discussion facilitated by the fitness instructor to promote shared experience and develop social connection.

### Self-management component

During this component participants exercised independently, following their HEP developed during the program. Participants received a 20-min video call every month from the fitness instructor, to review and adapt the HEP as required and to support problem solving to overcome any PA barriers. Full details of the PDConnect intervention are provided in the Supplemental Material.

### Usual care

Participants randomized to usual care received six, one-hour weekly sessions of physiotherapy. Usual care was delivered by community-based physiotherapists who had two or more-year post-registration experience of working in neurology or geriatrics but had not received specialist post-registration training in the management of PD. Each session included assessment, treatment, goal setting, intervention delivery, and provision of a HEP. Treatment choices were guided by participant need, as per standard practice including for example exercise prescription, education and advice. On completion of the 6-weeks participants were given advice and received no further intervention.

### Outcome measurement

The primary aim of this study was to determine the feasibility and acceptability of a multi-component intervention (PDConnect) aimed at promoting PA and self-management in community dwelling adults with PD, to inform a future large-scale effectiveness study.

### PDConnect intervention feasibility

Recruitment feasibility was determined by examining the time taken and number of participants screened, deemed eligible and enrolled. Retention rate was determined by calculating the percentage of participants who completed the intervention. Attendance was calculated as the proportion of sessions attended. Outcome measure and activity diary completion and return rates were determined by the proportion returned at each data collection point, and the number and type of adverse events were collated throughout the study. Following the CONSORT extension for feasibility and pilot studies,^37^ pre-specified progression criteria including participant recruitment, protocol adherence, and outcome data, were agreed *a priori* using the traffic light progression criteria system described by Avery et al.^43^ (see Supplemental Material). The go criteria for participant recruitment included 100% recruitment to target within 6 months, and less than 15% (n = 5) of participants withdrawing from the study. Go criteria for intervention fidelity was ≥85% of the intervention being delivered as planned. The go criteria for intervention included attending 5 out of the possible six 1:1 physiotherapy sessions and attending a minimum of 9 out of 12 of the group-based exercise sessions. Go criteria for return of outcome measures was between 80–100% of measures returned at each phase.

### PDConnect intervention fidelity

Fidelity assessment was conducted at the end of the study to explore what proportion of sessions adhered to ≥75% of PDConnect intended content. A random sample of video recordings from Microsoft Teams, representing 10% of PDConnect physiotherapy sessions and eight group-based exercise sessions were selected for fidelity assessment which were conducted by one researcher (JJ). Session plans were provided to staff prior to the start of the study to guide intervention delivery. Checklist were developed and mapped to these session plans allowing the researchers to assess intervention fidelity.

### PDConnect intervention acceptability

Intervention acceptability was explored using a survey and semi-structured interviews conducted at the end of the study. Satisfaction was assessed using an 11-point visual analogue scale (VAS), from zero (not at all satisfied) to 10 (very satisfied). Interviews were conducted by one researcher (EH) using Microsoft Teams™ following a standardized topic guide (Supplemental Material) to explore their perceptions of receiving and delivering PDConnect. In addition, the acceptability of the research approach was also explored, as the focus of this study was to inform the development of a future trial, therefore perceptions of being randomized and whether this may impact enrollment were deemed important. Interviews lasted up one hour. All interviews were audio recorded and intelligently transcribed by one researcher (JJ). Semi-structured interviews were also conducted with staff to explore their perceptions of intervention delivery.

Secondary outcomes were measured at baseline, and at six, eighteen, and thirty-weeks to provide preliminary data on outcomes such as PA, motor and non-motor symptoms, self-efficacy, and quality of life. A suite of measures were used to inform selection of measures for use in a future effectiveness study, and to calculate effect sizes to enable power calculations to be conducted.

*Motor function was measured by:* Unified Parkinson's Disease Rating Scale (UPDRS),^44^ Schwab and England Scale,^45^ and Activities-specific Balance Confidence Scale.^46^ All measures are recommended by the Movement Disorders Task Force which have been shown to be reliable and valid for PwP.^47,48^ In addition, individual physical activity (daily step count) was measured using a wrist-worn activity tracker (Mi band version 5.0). The Mi band has good internal consistency among health individuals during the six-min walk test and stairs climb (ICC: 0.830).^49^ Daily step counts were recorded within the activity diary. Self-reported PA was also recorded using the Physical Activity Scale for the Elderly.^50^ The Patient Global Impression of Change (PGIC) scale (11-point VAS) was used gauge participants perceptions of how participation had impacted on their perceived physical activity levels, knowledge and exercise confidence.

*Non-motor function was measured by:* UPDRS, Lille Apathy scale, Parkinson's Anxiety scale (PAS), Parkinson's Fatigue Scale (PFS), Geriatric Depression Scale (GDS), Parkinson's Disease Questionnaire 39 (PDQ39), and Nottingham Health Profile (NHP). All measures are recommended by the Movement Disorders Task Force and have established validity and reliability within PD properties.^51–53^ The Lille apathy scale (LARS) is a semi-structured interview that assesses apathy in nine domains and produces a global score ranging from −36 to +36, with higher scored indicating greater apathy.^54^ The PAS is a 12-item scale encompassing persistent and episodic anxiety, avoidance behavior.^55^ This scale uses a 5-pont Likert score, with a maximum score of 48, with higher scores indicative of greater anxiety. The PFS contains 16 items and is designed to assess the presence of fatigue and its impact on daily activities.^56^ A score of 16–80, based on the sum of scores for all 16 items is calculated with higher scores indicating higher levels of fatigue and great impact on daily life. The GDS is used to identify depression in older adults.^57^ Scores range from 0–30 with higher scores indicating more severe depression. The PDQ-39 is a PD-specific quality of life (QoL) measure, encompassing 39 questions, encompassing specific dimensions of functioning and well-being, with higher scores indicative of poorer QoL.^58^ NHP assess dimensions of health including pain, physical mobility, emotional reactions, energy, social isolation, and sleep.^59^ A total score for each domain is 100 where a score of 0 indicates good subjective health status and 100 indicates poor subjective health status.

The self-efficacy scale for exercise is a self-report measure that was used to establish participant exercise confidence.^60^ Participants rate their confidence on an 11-point VAS covering nine different items, with higher scores indicative of great exercise confidence. Finally, the Warwick Edinburgh Mental Health and Wellbeing Scale (WEMWBS) was used to assess mental wellbeing.^61^ The 14-item scale incorporates feeling and functioning aspects of mental wellbeing. The minimum score is 14 and maximum score of 70, with higher scores are indicative of greater mental health and wellbeing.

### Data analysis

Quantitative data including feasibility measures, demographic information, and baseline measures were analyzed in SPSS (V24) using descriptive statistics. As a feasibility study with a small sample size, test statistics for differences between groups were not calculated, due to the increased risk of type II error. In addition, they serve only to identify if an effect or difference exists, but do not allow inferences to be drawn on the size of the effect. Therefore, effect sizes were used to determine the sample size for potential future pilot RCTs, which aligned with a key aim of this research study. Cohen's *d* was used to estimate the size of effect, by analyzing the difference of mean change between PDConnect and usual care at the six, 18- and 30-weeks data collection timepoints for each measure. Cohen's *d* was interpreted as 0.2 (small), 0.5 (medium), and 0.8 (large) effect sizes.^62^

Qualitative data was analyzed by JJ and KC using Framework Analysis.^63^

## Results

### Feasibility

Participants were recruited between December 2020 and February 2021, and the study concluded in October 2021. The recruitment rate was 76%, with 31 participants enrolling out of 41 expressing an interest within 12 weeks, exceeding the go criteria for recruitment. Five people were excluded as they did not meet the study inclusion criteria, one person was excluded following screening as they did not wish to commit to a 30-week study for personal and social reasons. The remaining four contacted the researcher after the target sample was achieved. Recruitment and retention are summarized in Figure 1, and participant baseline characteristics are presented in Table 1. Staff recruitment took 10 weeks with an average six hours to complete the study specific training. No falls were reported when participating in exercise associated with PDConnect or usual care. One participant fractured their ankle and withdrew following a trip over garden furniture.

**Table 1.** Participant demographic and clinical characteristics at baseline.

Participant demographic and clinical characteristics at baseline	Total sample	PDConnect (n = 16)	Usual care (n = 15)
Age (Mean ± SD) years	66.4 ± 8.1	68.3 ± 8.3	64.6 ± 7.6
Sex	13 females (42%)	6 females (37%)	7 females (47%)
	18 males (58%)	10 males (63%)	8 males (53%)
Time since diagnosis (Mean ± SD) years	4.8 ± 2.6	4.5 ± 2.3	5.2 ± 2.9
Hoehn and Yahr Score Mode (range)	2.5 (1–3)	2.5 (1–3)	2.5 (1–3)
UPDRS (total) (Mean ± SD)	73.37 ± 16.14	72 00 ± 3.98	75.00 ± 18.53
UPDRS Part III (Mean ± SD)	31.24 ± 8.82	32.00 ± 8.87	30.43 ± 9.00
PDQ-39, (Median, IQR)	9.64,5.99–13.28	8.72, 4.85–13.79	10.42, 5.99–13.28
PASE (Mean ± SD)	139.57 ± 81.86	157.16 ± 80.60	120.84 ± 81.67

SD: standard deviation; IQR: interquartile range; UPDRS: Unified Parkinson's Disease Rating Scale; PDQ39: Parkinson's Disease Questionnaire 39; PASE: Physical Activity Scale for the Elderly.

Participant retention rate was 74% (n = 24), with an equal number of withdrawals from each group. Two participants withdrew within the first two-weeks of the study due to issues with technology. A further six participants withdrew due to health reasons unrelated to PD. Participant retention fell one percent below the amber threshold (75%) for progression to a full trial. Attendance and return of outcome measures reached the go progression criteria indicating proceed to a full trial. Intervention attendance was exceptional, with 100% participation in 1:1 Physiotherapy, with all participants attending all six sessions. Attendance remained high within the group-based component with participants attending 10 of the possible 12 the group-based exercise sessions (83% attendance). One participant did not return their outcome measures at the week 18 collection point; outcome measure return was 100% at all other time points. Most (84%) participants returned completed activity diaries at the end of the study. No adverse events were reported during intervention delivery or the HEP component. Additionally, all participants completed their monthly follow-up calls, successfully meeting the green (go) progression criteria.

### Intervention fidelity

Most physiotherapy sessions (89%) and group-based exercise sessions (88%) were delivered as planned. Mean time spent exercising during physiotherapy was 29 min (range 20–38 min). Lower exercise duration was moderated by participant preference, with many participants having completed their HEP prior to having physiotherapy that day. The mean duration of group-based exercise sessions was 63 min (range 58–66 min). 89% of 1:1 physiotherapy and 88% of group-based exercise sessions delivered as planned, meeting the go *a priori* progression criteria for this study.

### Intervention acceptability: quantitative findings

*Intervention delivery.* Satisfaction with PDConnect was high (see Table 2), with all 12 participants reporting that they would recommend PDConnect to other PwP. Staff were perceived as knowledgeable, caring, with strong communication skills.

**Table 2.** Satisfaction with PDConnect program.

Satisfaction domain	Visual analogue scale score (0–10)					
	10	9	8	7	6	<5
Satisfaction with Physiotherapy component of PDConnect	75% (n = 9)	0 (n = 0)	17% (n = 2)	8% (n = 1)	0 (n = 0)	0 (n = 0)
Satisfaction with the group-based exercise component of PDConnect	58% (n = 7)	25% (n = 3)	8% (n = 1)	0 (n = 0)	8% (n = 1)	0 (n = 0)
Physiotherapists knowledge of Parkinson's	83% (n = 10)	17% (n = 3)	0 (n = 0)	0 (n = 0)	0 (n = 0)	0 (n = 0)
Fitness Instructors knowledge of Parkinson's	67% (n = 8)	17% (n = 2)	8% (n = 1)	8% (n = 1)	0 (n = 0)	0 (n = 0)
Physiotherapists approachability	83% (n = 10)	17% (n = 3)	0 (n = 0)	0 (n = 0)	0 (n = 0)	0 (n = 0)
Fitness Instructors approachability	75% (n = 9)	17% (n = 2)	8% (n = 1)	0 (n = 0)	0 (n = 0)	0 (n = 0)
Physiotherapists communication skills	83% (n = 10)	8% (n = 1)	8% (n = 1)	0 (n = 0)	0 (n = 0)	0 (n = 0)
Fitness Instructors communication skills	75% (n = 9)	17% (n = 2)	8% (n = 1)	0 (n = 0)	0 (n = 0)	0 (n = 0)

Three-quarters of participants (n = 9) reported that the PDConnect manual was helpful or very helpful. The 30-week PDConnect intervention was deemed to be appropriate by all participants, however, 58% (n = 7) indicated that the 1:1 Physiotherapy component was too short, with the remainder indicating it was about right. Similarly, 58% (n = 7) perceived that the duration of the 12-week group component was about right, whereas 33% (n = 4) thought it was too short. Just over half (58%; n = 7) perceived that the self-management component was just right whereas 44% (n = 4) thought it was too short. No adverse incidents were reported during the study.

### Intervention acceptability: qualitative findings

14 PDConnect participants took part in semi structured interviews, including 2 participants who withdrew following completion of physiotherapy and a minimum of 75% of the group-based exercise. Three themes were identified from the Framework analysis: experience of the research process, experience of intervention delivery, and perceptions of intervention participation.

*Experience of the research process:* Information to support participation was deemed acceptable. Incorporation of randomization was perceived as a sign of robust research methodology, and participants expressed a sense of loyalty to the study once they had consented:

“*I thought randomization was a really important part of it, and I could completely understand why it [randomization] had been done… I was absolutely delighted that I was randomized to the bit I got, but yes absolutely having committed to participate I would have gone with whichever one I was chosen for*” (PDC52)

*Experience of PDConnect delivery.* Four subthemes were identified: online delivery, the pivotal role of staff, perceptions of PDConnect components and perceptions of PDConnect resources. Online delivery of PA was perceived to reduce many of the barriers associated with PA such as low motivation, transportation, and provided some participants with access to a specialist physiotherapist which they would otherwise not have had access to.

“*So, transport would have been a serious issue for me, and I wouldn't be able to do it, so I wouldn't have been a participant in this project if it had not been online*.” (PDC ID80)

“I was very impressed with the physiotherapist, and I found the very kind and helpful and you know she just took me through quite gently, considering all my needs not just my Parkinson's.” (PDC ID49)

However, challenges with Wi-Fi, initiating conversations, and positioning the digital device particularly for floor-based exercises were highlighted.

“*I didn't really enjoy the lying down ones [exercises], but that was purely because it was online and it was difficult to do the exercise, plus keep an eye on the screen.” (PDC ID 52)*

“*All the participants had slightly different internet connection experiences, so it didn't make for an easy discussion in the group.” (PDC ID91)*

Staff were perceived as pivotal to the success of the intervention, with participants valuing their personalized approach to delivery and motivation which inspired participants to be active.

“*She tailored the exercises to my abilities, couldn’t be better in relation to my needs… she took it on board what I said, and she focused on what was needed and met my requirements, the fitness instructor was much the same” (PDC ID1)*

“*I felt like she had knowledge far, far beyond what even I expected, for a fitness person, I never expected them to be clued up in Parkinson's, but I guess she must've done some preparation…the fitness instructor is remarkable, I mean she's had so much patience, so much enthusiasm, so much encouragement, all from a screen where she's like getting no feedback from us because we're all on mute. I am just in total awe that she was able to do that to be honest” (ID81).*

PDConnect was perceived as enjoyable with many participants expressing sadness when the intervention ceased. The weekly delivery was perceived to positively impact motivation to start and maintain being active and facilitated the initiation of a PA routine.

“*I think it was positive because it was a weekly meeting and that was the motivation… the motivation to keep doing it was the fact that the Physiotherapist was going to be there every week. The first six weeks were very positive, so at the end of that six weeks, I probably felt better than I had done for a wee while.”* (PDC ID51)

“*I mean, it could have went on longer, I think it could have went longer and because it was a very very beneficial!”* (PDC ID71)

Intervention resources were perceived as acceptable. Three quarters (75%) of the sample reported that the manual was helpful or very helpful, although differences in views existed reflecting individual preferences to know more about PD. Desire for an interactive dashboard to allow tailoring of information on PD and PA was suggested.

*Perceptions of intervention delivery.* Subthemes related to perceived benefits and challenges associated with participation in PDConnect. Physical, education and psychosocial benefits, highlighted below, were collectively perceived to positively influence PA behavior.

“*it was absolutely key to me to change my lifestyle at that time. It actually inspired me to look further for more exercise opportunities”* (PDC ID78)

“*It gave me a regime to try and stick to because over the period of time it showed me the benefits of it” (PDC ID71)*

“It brought some awareness too because I was quite active, but it showed me how important it was to kind of maintain that level of activity and also push myself from time to time. She got me back out on my bike again which I was a bit unconfident because I'd fallen off it before and stuff like that. So, she was good in an encouraging way to motivate you to try things out your comfort zone a little bit.” (PDC ID 91)

### Experience of physiotherapists and fitness instructors

The staff who delivered PDConnect and usual care were also interviewed. Two themes were identified from the Framework analysis: processes and resources involved in intervention delivery, and perceptions of delivering the intervention.

*Processes and resources involved in intervention delivery.* All staff reported benefits and challenges of using Microsoft Teams. Despite providing participants with a Teams induction staff reported it took participants time to become familiar and confident using Teams. However, these challenges were not unique nor PwP.

“*It worked very well, and I didn't have any significant challenges compared to other members of the public that was I working with online at the time.*” (Staff 001, Usual Care Physiotherapist).

Microsoft Teams presented different challenges during the group-based exercise component of PDConnect. Having up to eight participants on the screen made viewing and providing of feedback more challenging:

“*…In a group setting online and seeing small pictures of people on a screen, it was very difficult for me to ascertain whether or not they were doing the exercises correctly.”* (Staff 003, Fitness instructor).

Aligning the camera to adequately assess participants’ movements required patience and effective communication to enable participants to angle their device accordingly. Identification of issues and provision of feedback was perceived to take longer online compared with face-to-face, due to the limited view of a participant:

“*If I was in the clinic, I would have been able to put my hands on and worked that out really quickly. It probably took us a couple of sessions because I was doing it from observation and trying to instruct but we still worked it out.”* (Staff 002, PDConnect Physiotherapist)

Equally the benefits of online delivery were also reported by staff:

“*Online was brilliant and allowed a lot of flexibility in what we were doing. So, we were able to move around the house. You know, there was even one patient who had had issues with a specific chair -getting in and out of that, so we so we could take the laptop over and have a look at that.”* (Staff 001, Usual Care Physiotherapist)

“*Some of them don't have the ability to drive anymore, they were living on their own or their wives have got issues now and they can't drive them. So, I think online really works well because they're in the comfort of their own home.”* (Staff 003, Fitness Instructor)

*Perceptions of delivering the intervention.* PDConnect staff received training prior to delivery of the PDConnect intervention. PDConnect staff reported that the training manual provided a comprehensive guide, covering a wide range of topics pertinent to the study.

“*The training manual is far as I was concerned was very concise. It had a really good flow too. It's lots of information and from my point of view for me coming in as a sort of fitness strength and conditioning person, it gave me a really good insight into Parkinson's as an illness, and the limitations and abilities that I should be looking to expect.”* (Staff 003, Fitness Instructor)

The participant's manual was viewed by PDConnect staff as comprehensive covering the information in sufficient detail and in a manner that was accessible. However, future consideration of the to how the manual is presented was highlighted by staff to allow enough information to educate and motivate participants but not so much that it appeared off putting.

“*What was really great from a manual point of view, is that it taught the clients a lot about their condition. I empowered them through the information, and that was so important because they were not just a patient, they were somebody that is taking charge of what was happening to them and you know they then said, now, I understand why I'm doing it.”* (Staff 003, Fitness Instructor)

“*It's really hard because some people, they devour such a resource and other people, it's overwhelming.*” (Staff 002, PDConnect Physiotherapist)

### Secondary outcomes

*Perceived impact of participating in PDConnect:* 92% of participants strongly agreed or agreed that participation in PDConnect made them more confident with exercise, developed improved knowledge and understanding of the benefits of exercise, and PDConnect provided participants with strategies to stay active. Participants also reported that participation positively impacted motor aspects of their PD; however, self-reported pain, fatigue, and sleep levels were unchanged. Using a global patient impression of change scale, 60% (n = 7) of PDConnect participants reported that they were much improved compared to 9% (n = 1) in the usual care group.

*Motor function:* Mean weekly step count in both groups increased over the 30-week intervention in the usual care and PDConnect groups (1117 and 536 steps respectively). At baseline the usual care group were less active as measured by the PASE (Physical Activity Scale for the Elderly) compared to the PDConnect group, however this difference was not statistically significant. Both groups demonstrated improvement in PASE scores over the 30 weeks; however, as illustrated in Table 3, small effects were demonstrated. Change in mean differences in UPDRS I-II between baseline and 30 weeks indicated improvement with a large effect size in both groups. However, only the PDConnect group demonstrated improvements in all UPDRS subsections with small to large effects sizes also reported in UPDRS III and IV (see Table 3). Large effects sizes in Total UPDRS were evident in both groups suggesting improvement over time. Changes in NMS over the 30-week study were variable in both groups as shown in Table 4. Perceived depression as measured by the GDS improved in both groups, with small to medium effect sizes. Anxiety as measured by the PAS improved in both groups from baseline to six week and continued to stay low for the duration of the study.

**Table 3.** Mean difference and effect sizes between baseline and 30 weeks (pre and post intervention).

Measure	Pre (Mean ± SD)	Post (Mean ± SD)	Mean difference	Effect size Cohen's *d*	95% CI of effect size
PASE					
PDConnect	157.16 ± 80.60	172.74 ± 43.28	15.58	0.03	−0.53 to 0.45
Usual Care	120.84 ± 81.67	147.96 ± 55.25	27.12	0.02	0.52 to 0.46
UPDRS I					
PDConnect	16.56 ± 5.53	7.66 ± 3.14	−8.9	2.51	0.46 to 4.55
Usual Care	17.66 ± 3.97	9.90 ± 7.42	−7.76	1.37	0.18 to 2.57
UPDRS II					
PDConnect	22.75 ± 7.25	8.41 ± 5.05	−14.34	1.53	0.22 to 2.83
Usual Care	25.80 ± 7.25	11.18 ± 6.43	−14.62	1.80	0.29 to 3.30
UPDRS III					
PDConnect	32.00 ± 8.87	15.70 ± 4.16	−16.30	0.96	0.05 to 1.86
Usual Care	30.43 ± 9.00	23.90 ± 12.62	−6.53	−0.09	−0.59 to 0.40
UPDRS IV					
PDConnect	0.50 ± 0.52	1.25 ± 1.35	0.75	0.30	−0.21 to 0.90
Usual Care	1.13 ± 2.23	3.27 ± 2.57	2.14	0.03	−0.52 to 0.46
UPDRS total					
PDConnect	71.81 ± 13.98	33.95 ± 11.20	−37.86	1.98	0.33 to 3.62
Usual Care	75.03 ± 18.53	48.27 ± 25.56	−26.76	0.80	−0.01 to 1.60
GDS					
PDConnect	7.5 ± 3.8	6.6 ± 5.2	−0.9	0.43	−0.16 to 1.03
Usual Care	7.4 ± 5.3	1.1 ± 1.2	−6.3	4.01	0.8- to 7.23
WEMWBS					
PDConnect	47.18 ± 17.79	50.00 ± (8.73)	2.82	−0.17	−0.68 to 0.34
Usual Care	53.00 ± 8.64	52.81 ± 10.11	−0.19	−0.05	−0.54 to 0.44

SD: standard deviation; UPDRS: Unified Parkinson's Disease Rating Scale; PASE; Physical Activity Scale for the Elderly; WEMWBS: Warwick Edinburgh Mental Health and Well-being Scale; GDS: Geriatric Depression Scale.

**Table 4.** Impact of usual care and PDConnect on Parkinson's non-motor symptoms.

	Baseline		6 weeks		18 weeks		30 weeks	
Measure	PDConnect	Usual care	PDConnect	Usual care	PDConnect	Usual care	PDConnect	Usual care
LAS (Mean ± SD)	−26.81 ± 6.46	−28.14 ± 4.41	−28.68 ± 2.72	- 26.46 ± 5.41	- 29.46 ± 5.15	−28.84 ± 3.18	−30.91 ± 2.23	−28.36 ± 5.50
PFS (Median,IQR)	47.5, 33.7–57.5	38.0, 27.0–54.0	30.0, 29.0–48.0	32.0, 20.0–52.0	38.5, 32.7–47.5	36.0, 24.5–54.5	49.0, 32.0–57.5	32.0, 20.0–57.0
PAS (Median,IQR)	17.0, 14.0–25.5	21.0,18.0–26.0	2.0, 1.3–8.7	5.0, 2.5–9.5	3.5, 1.0–6.5	5.0, 4.0–12.0	5.5, 2.0–7.7	5.0, 0.0–13.0
GDS (Mean ± SD)	7.5 ± 3.8	7.4 ± 5.3	8.1 ± 4.7	5.2 ± 3.8	8.3 ± 5.0	6.2 ± 4.9	6.6 ± 5.2	1.1 ± 1.2

SD: standard deviation; IQR: interquartile range; LAS: Lille Apathy Scale; PFS: Parkinson's Fatigue Scale; PAS: Parkinson's Anxiety Scale; GDS: Geriatric Depression Scale.

## Discussion

This study aimed to establish the feasibility and acceptability of the PDConnect intervention along with effect size estimations for PA, motor, and non-motor symptoms, QoL, and health and well-being. The results demonstrate that online delivery of PDConnect is feasible and acceptable to PwP, and the study processes are feasible to conduct. Prior to scaling up to a large-scale RCT to test the effectiveness of PDConnect, minor modifications to study resources and outcome measures are indicated. All progression criteria, except participant retention, were met, with retention rate at 74%, one percent below the *a priori* progression criterion. Participant retention reported in the current study aligns with a recent systematic review of PA trials involving sedentary older people,^64^ and studies involving PwP.^8^ Low withdrawal rates combined with no adverse events and high acceptability would suggest that online delivery of PDConnect is a viable option for PwP and that a large scale RCT to test effectiveness of PDConnect is warranted.

Intervention fidelity was high in the current study (>85%). When assessing intervention fidelity, exercising for 35 min during physiotherapy sessions was the most frequently omitted item. This was attributed to participants’ preference to use appointment times to gain feedback on their exercise technique, having completed their exercise prior to the appointment. This was confirmed by the qualitative data. Opportunity for feedback and discussion was highly valued by participants and contributed to intervention satisfaction. Feedback has been shown to be integral to motor learning theory,^65^ supporting skill acquisition,^66^ and behavioral change.^34^ The value of feedback for learning has also been highlighted in bridging the gap between actual and desired performance.^67^ Learning has been shown to be optimized when feedback is frequent and based on direct observation of a task, facilitating self-reflection and problem solving.^67^ What cannot be determined from this study is whether feedback supported motor learning or whether the weekly structure of PDConnect provided accountability which positively impacted on learning.

The current study was not designed nor powered to explore effectiveness. However, the positive trends highlighted within the PDConnect group combined with the interview data, suggest that weekly feedback delivered over 18 weeks may have promoted consolidation of PA performance and development of confidence in participation in PA. Ellis et al. (2011) reported that self-efficacy is a key determinant of PA behavior,^68^ therefore the weekly nature of PDConnect may have allowed for development of greater self-confidence engaging in PA. Traditionally, research has focused on exercise effectiveness in terms of strength or balance gains. Findings from the current study would suggest that provision of feedback, mode of delivery and Intervention dose may be central to shaping PA performance and behavior, which needs further exploration in a suitably powered study.

PDConnect was perceived as highly acceptable, with high levels of satisfaction reported by participants and staff. This study demonstrates that online delivery of PDConnect is acceptable to support PwP to manage their PA, adding to the growing body of evidence supporting online delivery of PA.^65–67^ Undoubtedly, the acceptability of online delivery may have been reflective of the absence of face-to-face PA options at the time of the study, which may have biased responses. However, qualitative data indicated that participation in PA online was perceived as convenient, reducing barriers associated with transport, costs, and stigma, which is congruent with systematic review evidence,^27^ and more recent mixed methods studies,^69–72^ suggesting that COVID-19 was not the sole factor influencing acceptability.

In the current study, the convenience offered by online delivery mitigated issues with transport, rurality, distance, and dependency on others, promoting participant attendance. Convenience offered by online attendance has also been demonstrated in city-based studies. Over half of participants in the Engage-PD study, conducted in New York, reported that they would not have attended had it not been delivered online due to transportation challenges.^73^ This suggests online delivery serves to mitigate transport barriers for those living in both urban and rural areas. The convenience of online delivery was also recognized by staff in the current study, as PDConnect provided an opportunity for participants to access professionals with expertise in PD which would otherwise not have been available to them. Therefore, provision of online PA may offer a pragmatic solution, offering access to specialist services while also addressing potential differences in the care that people receive and the opportunities that they have to lead healthy lives.

Online delivery was not without its challenges. In the current study, digital literacy varied from participants first experience of video conferencing to highly competent. Online challenges were restricted to the initial weeks of the study, and were not unique to PwP, nor Microsoft Teams. Similar initial issues with Zoom were highlighted in the PDEx study,^72^ suggesting that regardless of the platform used, PwP may need additional time to develop digital literacy and confidence in using technology, which needs further consideration prior to a future trial.

The value of PD informed staff, and personalized care have been highlighted previously.^27,33^ In the current study, staff understanding of PD allowed development of tailored PA programs and the delivery of contextualized education promoting understanding of the purpose of prescribed PA. Participants felt ‘listened to’ with PA programs developed that addressed their PD and wider health and social needs, which may have contributed sustained improvement in anxiety reported in this study. Although based on a small sample, 50% of PDConnect participants reported feeling much improved following participation compared with 10% of usual care participants reiterating prior research which suggests that use of PD specialists may result in enhanced health outcomes.^74^ While prior research has illustrated the benefits of PA, this study would also suggest that the interventional dosage, opportunity for feedback, and attributes of staff are key ingredients to shape PA among PwP.

The current study's qualitative findings suggest that staff were integral to participant satisfaction. In the current study the physiotherapist and fitness instructors’ knowledge of PD was perceived to motivate, educate, and empower participants to be active. While no guidelines exist for Fitness instructors, the UK Parkinson's guidelines recommend that PwP should be seen by a PD specialist physiotherapist,^75^ and competencies exist for both fitness instructors and physiotherapists.^76^ However, a national audit highlighted a knowledge and skills gap in the current workforce.^77^ Similarly, an older Dutch survey of healthcare professionals reported that 75% lacked sufficient PD expertise.^78^ Although the current study was not designed nor powered to explore the impact of PD training, the findings provide preliminary evidence that 12 h of self-directed and practical-based learning may be sufficient to provide the necessary expertise. While NICE guidelines advocate that PwP should see a PD specialist physiotherapist, no consensus exists on what defines a PD physiotherapist.^75^ Further research is required to define what knowledge, skills and attributes are required of a specialist physiotherapist to inform the development of training programs to meet the gap in the current workforce.

As a feasibility study, several secondary measures were employed in the current study. Our small sample size prevents the drawing of specific inferences from the results, but they indicate that the UPDRSIII, PASE, and Global Patient Impression of Change Scale hold most promise for consideration within a future study. Power calculations based on participants step count were discounted owing to the variability in the reliability of this measure. For example, activities such as cycle and walking while pushing a buggy did not reliability measure step count. Global impression of change was also discounted as only those in the intervention arm of the study completed this measure in this study. Small to large effects sizes for the PASE and UPDRSIII suggest that participation in PDConnect may have positively impacted PA levels and PD motor symptoms. These findings align with prior research which suggest that regular participation in PA is associated with slower rate of decline for PwP.^25,79^ The UPDRS is regarded as the gold standard measure, but equally it is criticized for being symptom focused with less emphasis placed on the impact PD has on everyday life. Participants in the current study indicated that measures of PA, QoL and well-being were most important to them.

This study highlights that the sample size requirements for future definitive trials will be strongly influenced by the choice of the primary outcome measure. Given that the primary aim of PDConnect is to support PwP in becoming more active, using PASE as the primary outcome in a future trial would be a pragmatic choice. However, based on the data from this pilot study, the effect size difference between the intervention and usual care groups for PASE were very small, necessitating a very large sample size. Using standard sample size calculation software and effect size estimates derived from this pilot study, a definitive trial with PASE as the primary outcome would require approximately 274 participants per group to achieve sufficient statistical power (1-β = 0.8, α = 0.05). When considering more heterogeneous populations and interventions, required sample sizes could range between 548 and 1000 participants per group. In contrast, this pilot study found a large to very large effect size difference when using the UPDRS-III, which specifically assesses motor function impairment in PwP. With this more specific outcome measure, the observed effect size suggests that sample sizes typically feasible within exercise versus control trials (e.g.,30 to 50 participants per group) are likely to provide statistical power close to 1. While uncertainty in population effect sizes remains inherent in pilot studies, these findings emphasize the substantial differences in sample size requirements depending on the choice of the primary outcome measure.

### Limitations

This study adopted a pragmatic design exploring the feasibility and acceptability of a novel intervention; however, it was not without its limitations in relation to research processes and study design. Convenience sampling from one geographical area limited ethnic and cultural diversity and resulted in a sample that was younger than the mean age of diagnosis reported in incidence studies.^80^ To maintain participant safety during this online intervention, those with severe balance abnormalities and significant cognitive issues were excluded. Therefore, only PwP in Hoehn and Yahr stages I-III were included, which is not representative of all people living with PD. The PDConnect group had higher baseline PASE score compared to usual care group (157.16 v 120.84) suggesting higher baseline physical activity. This difference may have confounded the findings as PDConnect may have already been more motivated to exercise at baseline compared to the usual care group. PDConnect baseline PASE were also higher than normative values (144 for males and 112 for females) for people aged between 65 and 69. However, the large standard deviation in the PASE score suggests a wide variation within the sample. Despite higher baseline PASE scores, all PDConnect participants self-reported improved levels of PA following completion of the study.

Intervention acceptability and feasibility were assessed using bespoke individual semi-structured interviews, and via a study specific survey and checklist. Although this allowed the researchers to focus on the key aspects of PDConnect intervention, it recognized that these measures have not been subject to any reliability testing, therefore the stability, responsiveness, and predictive validity of the approaches used is unknown. In addition, the acceptability of usual care was not explored which is also a potential limitation of this study.

Intervention volume received by both study arms was not equal. The intervention arm received an additional 12 weeks of group exercise and monthly phone calls for three months. As the principal aim of this research was to determine the feasibility and acceptability of PDConnect rather than its effectiveness, the equity in volume was not a consideration for this study. In the current study usual care reflected the number of Physiotherapy sessions typically provided within the NHS (personal communication). A future RCT to investigate the effectiveness of PDConnect needs to address this inequity in intervention load. Owing to the nature of the intervention, participants could not be blinded, potentially impacting on the reliability of the findings.

### Conclusions

The current study adds to the growing evidence that online telehealth interventions can be used to support PwP to participate in PA. This study has demonstrated it is feasible to recruit to and deliver a multi-component online PA intervention, combining specialist physiotherapy and group-based exercise to support increased PA and PA self-management. We have identified amendments to be made to the intervention and study processes before a full evaluation can be conducted, namely: i) refining study manuals; ii) review of sampling strategy to ensure sample diversity; and iii) strategies to optimize digital engagement. In keeping with MRC guidance for the development of complex interventions,^36^ this will enable the effectiveness of PDConnect to be investigated in a large-scale study.

## Supplemental Material

sj-docx-1-pkn-10.1177_1877718X251324415 - Supplemental material for Feasibility and acceptability of PDConnect, a multi-component intervention to support physical activity in people with Parkinson's disease: A mixed methods study Supplemental material, sj-docx-1-pkn-10.1177_1877718X251324415 for Feasibility and acceptability of PDConnect, a multi-component intervention to support physical activity in people with Parkinson's disease: A mixed methods study by Julie Jones, Lyndsay Alexander, Elizabeth Hancock and Kay Cooper in Journal of Parkinson's Disease

## References

[1] Dorsey R Bloem B . Global, regional, and national burden of Parkinson’s disease, 1990–2016: a systematic analysis for the Global Burden of Disease Study 2016. Lancet Neurol 2018; 17: 939–953. 30287051 10.1016/S1474-4422(18)30295-3 PMC6191528

[2] Parkinson’s UK. Parkinson's UK Audit 2017, https://www.parkinsons.org.uk/sites/default/files/2018-05/Reference%20Report_2017.pdf (2017, accessed 7 February 2024).

[3] Ferrazzoli D Ortelli P Zivi I , et al. Efficacy of intensive multidisciplinary rehabilitation in Parkinson's disease: a randomised controlled study. J Neurol Neurosurg Psychiatry 2018; 89: 828–835. 29321141 10.1136/jnnp-2017-316437 PMC6204945

[4] Hechtner MC Vogt T Zöllner Y , et al. Quality of life in Parkinson's disease patients with motor fluctuations and dyskinesias in five European countries. Parkinsonism Relat Disord 2014; 20: 969–974. 24953743 10.1016/j.parkreldis.2014.06.001

[5] Chen H Zhao EJ Zhang W , et al. Meta-analyses on prevalence of selected Parkinson's nonmotor symptoms before and after diagnosis. Transl Neurodegener 2015; 4: 1. 25671103 10.1186/2047-9158-4-1 PMC4322463

[6] Hirsch MA van Wegen EEH Newman MA , et al. Exercise-induced increase in brain-derived neurotrophic factor in human Parkinson's disease: a systematic review and meta-analysis. Transl Neurodegener 2018; 7: 7. 29568518 10.1186/s40035-018-0112-1 PMC5859548

[7] Johansson ME Cameron IGM Van der Kolk NM , et al. Aerobic exercise alters brain function and structure in Parkinson's disease: a randomized controlled trial. Ann Neurol 2022; 91: 203–216. 34951063 10.1002/ana.26291 PMC9306840

[8] van der Kolk NM de Vries NM Kessels RPC , et al. Effectiveness of home-based and remotely supervised aerobic exercise in Parkinson's disease: a double-blind, randomised controlled trial. Lancet Neurol 2019; 18: 998–1008. 31521532 10.1016/S1474-4422(19)30285-6

[9] Ramazzina I Bernazzoli B Costantino C . Systematic review on strength training in Parkinson’s disease: an unsolved question. Clin Interv Aging 2017; 12: 619–628. 28408811 10.2147/CIA.S131903 PMC5384725

[10] da Silva FC Iop RDR de Oliveira LC , et al. Effects of physical exercise programs on cognitive function in Parkinson's disease patients: a systematic review of randomized controlled trials of the last 10 years. PLoS One 2018; 13: e0193113. 10.1371/journal.pone.0193113 PMC5828448 29486000

[11] de Oliveira MPB Lobato DFM Smaili SM , et al. Effect of aerobic exercise on functional capacity and quality of life in individuals with Parkinson's disease: a systematic review of randomized controlled trials. Arch Gerontol Geriatr 2021; 95: 104422. 33932826 10.1016/j.archger.2021.104422

[12] Chen K Tan Y Lu Y , et al. Effect of exercise on quality of life in Parkinson's disease: a systematic review and meta-analysis. Parkinsons Dis 2020; 2020: 3257623. 32695306 10.1155/2020/3257623 PMC7368221

[13] Cusso ME Donald KJ Khoo TK . The impact of physical activity on non-motor symptoms in Parkinson's disease: a systematic review. Front Med (Lausanne) 2016; 3: 35. 27583249 10.3389/fmed.2016.00035 PMC4987718

[14] Cristini J Weiss M De Las Heras B , et al. The effects of exercise on sleep quality in persons with Parkinson's disease: a systematic review with meta-analysis. Sleep Med Rev 2021; 55: 101384. 32987321 10.1016/j.smrv.2020.101384

[15] Wu PL Lee M Huang TT . Effectiveness of physical activity on patients with depression and Parkinson's disease: a systematic review. PLoS One 2017; 12: e0181515. 10.1371/journal.pone.0181515 PMC5531507 28749970

[16] Petzinger GM Fisher BE McEwen S , et al. Exercise-enhanced neuroplasticity targeting motor and cognitive circuitry in Parkinson's disease. Lancet Neurol 2013; 12: 716–726. 23769598 10.1016/S1474-4422(13)70123-6 PMC3690528

[17] Frazzitta G Balbi P Maestri R , et al. The beneficial role of intensive exercise on Parkinson disease progression. Am J Phys Med Rehabil 2013; 92: 523–532. 23552330 10.1097/PHM.0b013e31828cd254

[18] Ahlskog JE . Does vigorous exercise have a neuroprotective effect in Parkinson disease? Neurology 2011; 77: 288–294. 21768599 10.1212/WNL.0b013e318225ab66 PMC3136051

[19] Mak MK Wong-Yu IS Shen X , et al. Long-term effects of exercise and physical therapy in people with Parkinson disease. Nat Rev Neurol 2017; 13: 689–703. 29027544 10.1038/nrneurol.2017.128

[20] Lauze M Daneault JF Duval C . The effects of physical activity in Parkinson’s disease: a review. J Parkinsons Dis 2016; 6: 685–698. 27567884 10.3233/JPD-160790 PMC5088404

[21] van Nimwegen M Speelman AD Hofman-van Rossum EJ , et al. Physical inactivity in Parkinson's disease. J Neurol 2011; 258: 2214–2221. 21614433 10.1007/s00415-011-6097-7 PMC3225631

[22] Mantri S Wood S Duda JE , et al. Comparing self-reported and objective monitoring of physical activity in Parkinson disease. Parkinsonism Relat Disord 2019; 67: 56–59. 31621608 10.1016/j.parkreldis.2019.09.004

[23] Paillard T Rolland Y De Barreto P . Protective effects of physical exercise in Alzheimer’s disease and Parkinson’s disease: a narrative review. J Clin Neurol 2015; 11: 212–219. 26174783 10.3988/jcn.2015.11.3.212 PMC4507374

[24] Klamroth S Steib S Devan S , et al. Effects of exercise therapy on postural instability in Parkinson disease: a meta-analysis. J Neurol Phys Ther 2016; 40: 3–14. 26655098 10.1097/NPT.0000000000000117

[25] Rafferty MR Schmidt PN Luo ST , et al. Regular exercise, quality of life, and mobility in Parkinson's disease: a longitudinal analysis of National Parkinson Foundation Quality Improvement Initiative Data. J Parkinsons Dis 2017; 7: 193–202. 27858719 10.3233/JPD-160912 PMC5482526

[26] Canning CG Sherrington C Lord SR , et al. Exercise for falls prevention in Parkinson disease: a randomized controlled trial. Neurology 2015; 84: 304–312. 25552576 10.1212/WNL.0000000000001155 PMC4335992

[27] Hunter H Lovegrove C Haas B , et al. Experiences of people with Parkinson's disease and their views on physical activity interventions: a qualitative systematic review. JBI Database Syst Rev Implement Rep 2019; 17: 548–613. 10.11124/JBISRIR-2017-003901 30973527

[28] Ellis T Rochester L . Mobilizing Parkinson’s disease: the future of exercise. J Parkinsons Dis 2018; 8: S95–S100. 10.3233/JPD-181489 PMC6311359 30584167

[29] Collett J Franssen M Meaney A , et al. Phase II randomised controlled trial of a 6-month self-managed community exercise programme for people with Parkinson's disease. J Neurol Neurosurg Psychiatry 2017; 88: 204–211. 27837101 10.1136/jnnp-2016-314508

[30] Hulbert SM Goodwin VA . ‘Mind the gap’—a scoping review of long term, physical, self-management in Parkinson’s. Physiotherapy 2020; 107: 88–99. 32026840 10.1016/j.physio.2019.12.003

[31] Tuijt R Tan A Armstrong M , et al. Self-management components as experienced by people with Parkinson's disease and their carers: a systematic review and synthesis of the qualitative literature. Parkinsons Dis 2020; 2020: 8857385. 33489082 10.1155/2020/8857385 PMC7787805

[32] Pigott JS Kane E Ambler G , et al. Systematic review and meta-analysis of clinical effectiveness of self-management interventions in Parkinson's disease. BMC Geriatr 2022; 22: 45. 35016613 10.1186/s12877-021-02656-2 PMC8753859

[33] Vlaanderen FP Rompen L Munneke M , et al. The voice of the Parkinson customer. J Parkinsons Dis 2019; 9: 197–201. 30373962 10.3233/JPD-181431

[34] Michie S Van Stralen MM West R . The behaviour change wheel: a new method for characterising and designing behaviour change interventions. Implement Sci 2011; 6: 42. 21513547 10.1186/1748-5908-6-42 PMC3096582

[35] Michie S Richardson M Johnston M , et al. The behaviour change technique taxonomy (v1) of 93 hierarchically clustered techniques: building an international consensus for the reporting of behaviour change interventions. Ann Behav Med 2013; 46: 81–95. 23512568 10.1007/s12160-013-9486-6

[36] Skivington K Matthews L Simpson SA , et al. A new framework for developing and evaluating complex interventions: update of medical research council guidance. BMJ 2021; 374: n2061. 10.1136/bmj.n2061 PMC8482308 34593508

[37] Eldridge SM Lancaster GA Campbell MJ , et al. CONSORT 2010 Statement: extension to randomised pilot and feasibility trials. BMJ 2016; 355: i5239. 10.1136/bmj.i5239 PMC5076380 27777223

[38] Sekon M Cartwright M Francis JJ . Acceptability of healthcare interventions: an overview of reviews and development of a theoretical framework. BMC Health Serv Res 2017; 17: 88. 28126032 10.1186/s12913-017-2031-8 PMC5267473

[39] Gearing RE El-Bassel N Ghesquiere A , et al. Major ingredients of fidelity: a review and scientific guide to improving quality of intervention research implementation. Clin Psychol Rev 2011; 31: 79–88. 21130938 10.1016/j.cpr.2010.09.007

[40] Borrelli B . The assessment, monitoring, and enhancement of treatment fidelity in public health clinical trials. J Public Health Dent 2011; 71: S52–S63. 10.1111/j.1752-7325.2011.00233.x PMC3074245 21499543

[41] Osborne JA Botkin R Colon-Semenza C , et al. Physical therapist management of Parkinson disease: a clinical practice guideline from the American Physical Therapy Association. Phys Ther 2022; 102: pzab302. 10.1093/ptj/pzab302 PMC9046970 34963139

[42] Keus SHJ Munneke M Graziano M , et al. European guidelines for physiotherapy in Parkinson’s disease, http://icfmobile.orgViewproject (2014, accessed 7 February 2024).

[43] Avery KN Williamson PR Gamble C , et al. Informing efficient randomised controlled trials: exploration of challenges in developing progression criteria for internal pilot studies. BMJ Open 2017; 7: e013537. 10.1136/bmjopen-2016-013537 PMC5318608 28213598

[44] Movement Disorder Society Task Force on Rating Scales for Parkinson's Disease. The Unified Parkinson's Disease Rating Scale (UPDRS): status and recommendations. Mov Disord 2003; 18: 738–750. 12815652 10.1002/mds.10473

[45] Schwab RS England ACJ . Projection technique for evaluating surgery in Parkinson’s disease. In: Gillingham FJ and Donaldson IML (eds) Third symposium of Parkinson’s disease. Edinburgh, Scotland: E&S Livingstone, 1969, pp.152–157.

[46] Powell LE Myers AM . The activities-specific balance confidence (ABC) scale. J Gerontol A Biol Sci Med Sci 1995; 50A: M28–M34. 10.1093/gerona/50a.1.m28 7814786

[47] Bloem BR Marinus J Almeida Q , et al. Measurement instruments to assess posture, gait, and balance in Parkinson's disease: critique and recommendations. Mov Disord 2016; 31: 1342–1355. 26945525 10.1002/mds.26572

[48] Shulman LM Armstrong M Ellis T , et al. Disability rating scales in Parkinson's disease: critique and recommendations. Mov Disord 2016; 31: 1455–1465. 27193358 10.1002/mds.26649

[49] Paradiso C Colino F Liu S . The validity and reliability of the Mi Band wearable device for measuring steps and heart rate. Int J Exerc Sci 2020; 13: 689–701. 32509127 10.70252/NJHQ9420 PMC7241628

[50] Washburn RA McAuley E Katula J , et al. The Physical Activity Scale for the Elderly (PASE): evidence for validity. J Clin Epidemiol 1999; 52: 643–651. 10391658 10.1016/s0895-4356(99)00049-9

[51] Leentjens AF Dujardin K Marsh L , et al. Apathy and anhedonia rating scales in Parkinson's disease: critique and recommendations. Mov Disord 2008; 23: 2004–2014. 18709683 10.1002/mds.22229

[52] Leentjens AF Dujardin K Marsh L , et al. Anxiety rating scales in Parkinson's disease: critique and recommendations. Mov Disord 2008; 23: 2015–2025. 18792121 10.1002/mds.22233

[53] Schrag A Barone P Brown RG , et al. Depression rating scales in Parkinson's disease: critique and recommendations. Mov Disord 2007; 22: 1077–1092. 17394234 10.1002/mds.21333 PMC2040268

[54] Sockeel P Dujardin K Devos D , et al. The Lille apathy rating scale (LARS), a new instrument for detecting and quantifying apathy: validation in Parkinson's disease. J Neurol Neurosurg Psychiatry 2006; 77: 579–584. 16614016 10.1136/jnnp.2005.075929 PMC2117430

[55] Leentjens AF Dujardin K Pontone GM , et al. The Parkinson Anxiety Scale (PAS): development and validation of a new anxiety scale. Mov Disord 2014; 29: 1035–1043. 24862344 10.1002/mds.25919

[56] Brown RG Dittner A Findley L , et al. The Parkinson fatigue scale. Parkinsonism Relat Disord 2005; 11: 49–55. 15619463 10.1016/j.parkreldis.2004.07.007

[57] Yesavage JA Brink TL Rose TL , et al. Development and validation of a geriatric depression screening scale: a preliminary report. J Psychiatr Res 1982; 17: 37–49. 7183759 10.1016/0022-3956(82)90033-4

[58] Jenkinson C Fitzpatrick R Peto V , et al. The Parkinson's Disease Questionnaire (PDQ-39): development and validation of a Parkinson's disease summary index score. Age Ageing 1997; 26: 353–357. 9351479 10.1093/ageing/26.5.353

[59] Hunt SM McEwen J McKenna SP . Measuring health status: a new tool for clinicians and epidemiologists. J R Coll Gen Pract 1985; 35: 185–188. 3989783 PMC1960139

[60] Resnick B Jenkins LS . Testing the reliability and validity of the Self-Efficacy for Exercise scale. Nurs Res 2000; 49: 154–159. 10882320 10.1097/00006199-200005000-00007

[61] Tennant R Hiller L Fishwick R , et al. The Warwick-Edinburgh Mental Well-being Scale (WEMWBS): development and UK validation. Health Qual Life Outcomes 2007; 5: 63. 18042300 10.1186/1477-7525-5-63 PMC2222612

[62] Cohen J . Statistical power analysis for the behavioral sciences. 2nd ed. New York: Routledge, 2013.

[63] Ritchie J Lewis J . Qualitative research practice: a guide for social science students and researchers. London: Sage, 2003.

[64] Howlett N Trivedi D Troop NA , et al. Are physical activity interventions for healthy inactive adults effective in promoting behaviour change and maintenance, and which behaviour change techniques are effective? A systematic review and meta-analysis. Transl Behav Med 2019; 9: 147–157. 29506209 10.1093/tbm/iby010 PMC6305562

[65] Kwon YH Kwon JW Lee MH . Effectiveness of motor sequential learning according to practice schedules in healthy adults; distributed practice versus massed practice. J Phys Ther Sci 2015; 27: 769–772. 25931727 10.1589/jpts.27.769 PMC4395711

[66] Winstein C Wing AM Whitall J . Motor control and learning principles for rehabilitation of upper limb movements after brain injury. Handb Neuropsychol 2003; 9: 79–138.

[67] Burgess A van Diggle C Roberts C , et al. Feedback in the clinical setting. BMC Med Educ 2020; 20: 460. 33272265 10.1186/s12909-020-02280-5 PMC7712594

[68] Ellis T Cavanaugh JT Earhart GM , et al. Factors associated with exercise behaviour in people with Parkinson disease. Phys Ther 2011; 91: 1838–1848. 22003171 10.2522/ptj.20100390 PMC3229047

[69] Lai B Bond K Kim Y , et al. Exploring the uptake and implementation of tele-monitored home-exercise programmes in adults with Parkinson's disease: a mixed-methods pilot study. J Telemed Telecare 2020; 26: 53–63. 30134777 10.1177/1357633X18794315

[70] Shah R Read J Davies N , et al. People with Parkinson's perspectives and experiences of self-management: qualitative findings from a UK study. PLoS One 2022; 17: e0273428. 10.1371/journal.pone.0273428 PMC9462566 36083947

[71] Larson D Yeh C Rafferty M , et al. High satisfaction and improved quality of life with Rock Steady Boxing in Parkinson's disease: results of a large-scale survey. Disabil Rehabil 2022; 44: 6034–6041. 34498995 10.1080/09638288.2021.1963854

[72] Bennett HB Walter CS Oholendt CK , et al. Views of in-person and virtual group exercise before and during the pandemic in people with Parkinson disease. PM R 2023; 15: 772–779. 35596118 10.1002/pmrj.12848 PMC10119971

[73] Shih HS Macpherson CE King M , et al. Physical activity coaching via telehealth for people with Parkinson disease: a cohort study. J Neurol Phys Ther 2022; 46: 240–250. 36170256 10.1097/NPT.0000000000000410

[74] Ypinga JHL de Vries NM Boonen LHHM , et al. Effectiveness and costs of specialised physiotherapy given via ParkinsonNet: a retrospective analysis of medical claims data. Lancet Neurol 2018; 17: 153–161. 29246470 10.1016/S1474-4422(17)30406-4

[75] National Institute for Health and Care Excellence. Parkinson’s Disease in adults. NICE guideline NG71, https://www.nice.org.uk/guidance/ng71 (2017, accessed 7 February 2024).

[76] Rafferty MR Hoffman L Feeney M , et al. Parallel development of Parkinson's-specific competencies for exercise professionals and criteria for exercise education programs. Parkinsonism Relat Disord 2023; 112: 105407. 37202275 10.1016/j.parkreldis.2023.105407

[77] Parkinson’s UK. Parkinson’s UK Audit Summary, https://www.parkinsons.org.uk/sites/default/files/202001/CS3524%20Parkinson%27s%20UK%20Audit%20-%20Summary%20Report%202019%20%281%29.pdf (2019, accessed April 2024).

[78] Nijkrake MJ Keus SH Oostendorp RA , et al. Allied health care in Parkinson's disease: referral, consultation, and professional expertise. Mov Disord 2009; 24: 282–286. 19170189 10.1002/mds.22377

[79] Amara AW Chahine L Seedorff N , et al. Self-reported physical activity levels and clinical progression in early Parkinson's disease. Parkinsonism Relat Disord 2019; 61: 118–125. 30554993 10.1016/j.parkreldis.2018.11.006

[80] Macleod AD Henery R Nwajiugo PC , et al. Age-related selection bias in Parkinson's disease research: are we recruiting the right participants? Parkinsonism Relat Disord 2018; 55: 128–133. 29871791 10.1016/j.parkreldis.2018.05.027

