# Supplementary material for: Feasibility and acceptability of PDConnect, a multi-component intervention to support physical activity in people with Parkinson's disease: A mixed methods study
Source: J Parkinsons Dis. 2025 Mar 28:1877718X251324415. Online ahead of print. doi: 10.1177/1877718X251324415 (PMC13347447; doi:10.1177/1877718X251324415)
Supplement: Supplementary file 1 [file sj-docx-1-pkn-10.1177_1877718X251324415.docx]

**Supplemental Material**

**Feasibility and acceptability of PDConnect, a multi-component intervention to support physical activity in people with Parkinson's disease: A mixed methods study**

**TIDieR (Template for Intervention Description and Replication) Checklist^1^**

|  | **TIDiER checklist domain** | **Content** |
| --- | --- | --- |
| 1 | Provide the name or a phrase that describes the intervention | PDConnect Intervention  PDConnect is an evidence-informed physical activity (PA) intervention aimed at providing people with Parkinson’s (PwP) with a toolkit of behavior change techniques to promote PA participation, and PA self-management. |
| 2 | WHY: Describe any rationale, theory, or goal of the elements essential to the intervention. | Research suggests many PwP are aware of the benefits of PA, yet many remain inactive. Physiotherapy has been shown to be effective, but PA adherence declines when physiotherapy ceases. Therefore, interventions are required which aim to support PwP develop long term PA habits to enable them to self-manage their PA are required.  PDConnect is evidence informed intervention:   - PA prescription is based on the European^2^ and American Physical Therapy Parkinson’s guidelines.^3^ - The COM-B model of behavioral changes and the Behaviour change Techniques taxonomy described by Michie et al.^4,5^ were embedded within the intervention. - Education provided within the intervention was informed by systematic review findings^6^ which highlighted what PwP motivated them to be active. |
| 3 | WHAT: Materials: Describe any physical or informational materials used in the intervention, including those provided to participants or used in intervention delivery or in training of intervention providers. Provide information on where the materials can be accessed. | - All participants received a manual providing guidance on the use of Microsoft Teams and how to use their physical activity tracker provided for use during the study. - An additional manual was provided to participants randomized to receive PDConnect to reinforce key components of the intervention and serve as an educational resource. Education included Parkinson’s pathophysiology, benefits of activity, types of PA, behavior change, health and safety, and tips for getting started and staying active. - Physiotherapists and Fitness Instructors delivering PDConnect received specialist training developed by the researcher prior to delivery. Training content was informed by current frameworks,^7^ Physiotherapy guidelines,^2,3^ and stakeholder consultation. - The staff training manual was divided into the following sections: Self-study guide; understanding Parkinson’s; medical management of Parkinson’s; the assessment of PwP; prescribing PA for PwP; promoting self-management; supporting behavior change; developing effective patient relationships; the PDConnect Intervention; using RehabGuru™; using Microsoft Teams and Mi bands. - Manual can be accessed via the corresponding Author. |
| 4 | Procedures: Describe each of the procedures, activities, and/or processes used in the intervention, including any enabling or support activities | **Usual care:** Participants received standard physiotherapy, and a home exercise program tailored to their individual needs.  **PDConnect:**  **1:1 Physiotherapy:** Each one-hour session comprised of individualized exercise prescription delivered in conjunction with education, self-management strategies, and BCTs, using a coaching style of delivery. A PDConnect manual was provided to reinforce key components of the intervention and serve as an educational resource. Education included Parkinson’s pathophysiology, benefits of activity, types of PA, behavior change, health and safety, and tips for getting started and staying active. BCTs were selected from the Behaviour Change Taxonomy described by Michie et al. (2013),^5^ and included goal setting, shaping knowledge, problem solving, developing self-awareness, self-belief, reward, and regulation. BCTs were mapped to each session to support development of PA self-management.  Individual session plans guided weekly content that included a minimum of 35-minutes of PA encompassing strength, balance, aerobic, flexibility, cognitive, gait, and functional components, with a focus on quality and amplitude of movement, in dual and single task activities as per current guidelines.^3^ Participants were encouraged to work at moderate to high intensity, defined as “somewhat hard” on the Borg Rating of Perceived Exertion scale.^8^ Exercises were mutually selected and delivered in tandem with education, to promote understanding, motivation, and adherence. Exercise was progressed on an individual basis, following the European Physiotherapy Guideline for Parkinson’s by increasing repetitions, speed, load, or task complexity.^2^ One-to-one sessions were supplemented with a home exercise plan (HEP), tailored to individual’s need and goals. HEPs were developed using REHABGuru® exercise library. Participants were encouraged to complete their HEP for 30-minutes, five times a week, and record all activity within an activity diary. Development of a PA routine was promoted within each session using an activity planner, self-completed activity diaries and self-monitoring of PA using an activity tracker. Development of routine was also promoted through weekly discussion reflecting on prior week activity, and the adoption of a problem-solving approach to address barriers or challenges which were encountered.  **Group-based exercise**: Each session included a minimum of 60-minutes exercise adopting a circuit-based approach. Exercise stations included mobility, strengthening, aerobic, balance, cognitive, and goal-oriented components with an emphasis on large amplitude movements and intensity of effort as recommended by current guidelines,^3^ and had four levels of difficulty, allowing tailoring to individual ability. Participants were provided with key teaching points, practical demonstration, and proposed benefits of each exercise. Videos of each station and individual levels were provided on Teams prior to participating. Throughout the class, the fitness instructor provided feedback to refine participant technique and progress as appropriate. Exercise was followed by 30-minutes of group-based discussion facilitated by the fitness instructor to promote shared experience and develop social connection.  **Self-management component:** During this component participants exercised independently, following the HEP which had been developed during the program. Participants received a 20-minute video call every month from the fitness instructor, to review and adapt the HEP as required and to support problem solving to overcome any PA barriers.  **Usual Care:** participants received six, one-hour long physiotherapy sessions delivered once a week for six weeks. Physiotherapy was delivered by a Physiotherapist who had received no post-registration training in Parkinson’s. an individualized program of physiotherapy was delivered based on the participants need including tailored exercise program, education, and self-management guidance. |
| 5 | WHO PROVIDED: For each category of intervention provider, describe their expertise, background and any specific training given. | **Physiotherapist eligibility criteria**: UK band 6 physiotherapist were required to be Band 6 or above, with a minimum of two years clinical experience, possessing a broad range of experience encompassing Neurology and Geriatrics. Band 6 Physiotherapists were selected as they are independent and autonomous practitioners, with an ability to modify assessments and treatments to meet individual need, with established communication skills.^9^  **Usual care physiotherapist**: Qualified Physiotherapist with 3 years of clinical experience, working with a broad range of patients. No specific training was provided as per the study protocol.  **PDConnect Physiotherapist:** qualified Physiotherapist with over 5 years’ experience, working with a broad range of patients including neurology. Received 12 hours of directed Parkinson’s specific training via a manual supported by a one-day practical course delivered via Microsoft Teams.  **Fitness instructor** **eligibility criteria**: Level three personal training qualification or above, which is Register of Exercise Professionals (REPS) accredited or equivalent. Level three or above reflects normal practice within local leisure facilities and ensured instructors had broad skills and experience in tailoring training sessions out with athletic populations.  **PDConnect Fitness instructor:** over 5 years’ experience as a personal trainer with experience in cardiac rehabilitation, musculoskeletal rehabilitation, athletic training, Pilates, yoga, and coaching. Received training as per the PDConnect Physiotherapist |
| 6 | Describe the modes of delivery of the intervention and whether it was provided individually or in a group. | **Usual Care:** delivered 6 weekly hour long 1:1 appointments delivered via Microsoft Teams  **PDConnect:** The intervention consists of three components: i) six sessions of one-to-one specialist physiotherapy; ii) 12 weekly sessions of group-based PA; iii) 12 weeks of self-management, with monthly telephone contact.  Both usual care and PDConnect were delivered using Microsoft Teams |
| 7 | WHERE: Describe the type(s) of location(s) where the intervention occurred, including any necessary infrastructure or relevant features. | The intervention was delivered in the participants own homes. All participants completed a home risk assessment and received a Microsoft Teams® induction prior to commencing the study. |
| 8 | WHEN AND HOW MUCH: Describe the number of times the intervention was delivered and over what period of time including the number of sessions, their schedule, and their duration, intensity or dose. | **Usual Care:** received 6 one-hour weekly sessions of 1:1 Physiotherapy and received a home exercise program tailored to their individual needs.  **PDConnect:**  **1:1 Physiotherapy:** six sessions of one-to-one specialist physiotherapy over a 6-week period. Individual session plans guided weekly content that included a minimum of 35-minutes of PA encompassing strength, balance, aerobic, flexibility, cognitive, gait, and functional components, with a focus on quality and amplitude of movement, in dual and single task activities. Participants were provided with a tailored home exercise program to undertake five times a week, with each session lasting a minimum of 30 minutes.  **Group-based exercise**: Sessions lasted 90 minutes with a minimum 60 minutes of PA, and 30 minutes for group education discussion, for 12 consecutive weeks. Exercise was delivered via a circuit-based approach. Participants spent four minutes at each station to allow sufficient time for participants to optimize performance within each station, recognizing that cognitive processing and motor learning can be slower among PwP.^10^ Exercise stations included mobility, strengthening, aerobic, balance, cognitive, and goal-oriented components with an emphasis on large amplitude movements and intensity of effort. Each station had four levels of difficulty, allowing tailoring to individual participant ability. Difficulty was progressed by the inclusion of a dual task or a cognitive challenge. |
| 9 | TAILORING: If the intervention was planned to be personalised, titrated or adapted, then describe what, why, when, and how. | Both usual care and PDConnect 1:1 physiotherapy was tailored to individual need, based on the physiotherapists assessment and participants preferences.  With the group-based component – tailoring of exercise was addressed by having had four levels of difficulty within each exercise station, allowing tailoring to individual participant ability. Difficulty was progressed by the inclusion of a dual task or a cognitive challenge. |
| 10 | MODIFICATIONS: If the intervention was modified during the study, describe the changes | No modifications were made during the study |
| 11 | Planned: If intervention adherence or fidelity was assessed, describe how and by whom, and if any strategies were used to maintain or improve fidelity, describe them. | Intervention adherence was assessed by having a register of attendance each week. This register was maintained by staff delivering the intervention.  Fidelity was explored used a mixed methods approach. Following completion of delivery semi structured interviews with staff were conducted to explore staff perceptions of intervention fidelity and explore factors which may have influenced fidelity. Intervention fidelity was also explored quantitatively using checklists. All PDConnect sessions were recorded using Microsoft Teams. Fidelity assessment was conducted by the researcher following completion of the study, after researcher un-blinding had occurred. Fidelity assessment was conducted to establish whether the intervention was delivered as planned, specifically content, delivery, and duration. Fidelity assessment was conducted by the researcher using a checklist mapped to the individual session plans provided to the Physiotherapists and Fitness Instructors. |
| 12 | Actual: If intervention adherence or fidelity was assessed, describe the extent to which the intervention was delivered as planned. | **Intervention attendance:** Attendance at 1:1 Physiotherapy for both the usual care and intervention groups was 100%. The group-based exercise attendance rate was 84%, with participants attending on average 10 out of 12 group-based sessions.  **Intervention fidelity:** On average 89% of components were delivered as intended (range 75% to 96%) |

**References**

1. Hoffmann TC, Glasziou PP, Boutron I, et al. Better reporting of interventions: template for intervention description and replication (TIDieR) checklist and guide. *BMJ* 2014; 348: g1687.
2. Keus SHJ, Munneke M, Graziano M, et al. European guidelines for physiotherapy in Parkinson’s disease. http://icfmobile.orgViewproject (2014, accessed 7 February 2024).
3. Osborne JA, Botkin R, Colon-Semenza C, et al. Physical therapist management of Parkinson disease: A clinical practice guideline from the American Physical Therapy Association. *Phys Ther* 2022; 102: pzab302.
4. Michie S, Van Stralen MM and West R. The behaviour change wheel: A new method for characterising and designing behaviour change interventions. *Implement Sci* 2011; 6: 42.
5. Michie S, Richardson M, Johnston M, et al. The behaviour change technique taxonomy (v1) of 93 hierarchically clustered techniques: building an international consensus for the reporting of behaviour change interventions. *Ann Behav Med* 2013; 46: 81–95.
6. Hunter H, Lovegrove C, Haas B, et al. Experiences of people with Parkinson's disease and their views on physical activity interventions: a qualitative systematic review. *JBI Database Syst Rev Implement Rep* 2019; 17: 548–613.
7. Allied Health Professions Competency Framework for Neurological Conditions, 2018. https://www.rcot.co.uk/sites/default/files/Competency%20framework%20-%20FINAL%20COPY.pdf.
8. Borg GA. Psychophysical bases of perceived exertion. *Med Sci Sports Exerc* 1982; 14: 377-381.
9. The Chartered Society Of Physiotherapy. Physiotherapy Framework. https://www.csp.org.uk/professional-clinical/cpd-education/professional-development/professional-frameworks (2018).
10. Pang SY, Ho PW, Liu HF, et al. The interplay of aging, genetics and environmental factors in the pathogenesis of Parkinson's disease. *Transl Neurodegener* 2019; 8: 23.

**Participant semi structure interview topic guide**

| **Areas of interest within Semi Structured Interviews** | **Probes/prompts to be used depending on how the answer the opening question:** |
| --- | --- |
| **Study Recruitment, we are interested to hear about your experience of being recruited to be involved in this study** | |
| - How did you find out about the study? What did you think of the participant information sheet that you were provided with? - This study involved participants being randomized to receive PDConnect or usual care which consisted of 6 sessions of physiotherapy. How did you feel about be randomized as part of this study? | - PIS -was it too much info/not enough/just, right? is there any other information that should have been included? - Would you still have taken part if you had been randomized to receive usual care? And why? |
| **Satisfaction with and experiences and perceptions of the PDConnect Intervention** | |
| **1:1 Physiotherapy:** we would now like to focus on the 6 sessions of 1-1 physiotherapy part of PDConnect, that was delivered by the physiotherapist   - Can you tell me what you thought of this part of the intervention? - The aim of 1:1 Physiotherapy was to develop your confidence with exercise, increase your awareness of the benefits of exercise, promote increased physical activity, and to develop an exercise program that meets your personal needs. - What are your perceptions, do you feel that the 1:1 physiotherapy achieved this or not? - If yes, tell me about your experience, if no, how does the intervention need to be changed? | - What were their thoughts on the duration, content and delivery? - Which elements of the 1:1 physiotherapy did you find the most and least beneficial - Was there anything you felt was missing from the 1:1 element of the program or is there anything you think should be removed. - Do you have any further comments that you wish to share about the 1:1 physiotherapy component? |
| **Group-based component:** We now want to focus on the 12 sessions of group-based exercise part of PDConnect, that was delivered by the fitness instructor.   - Can you tell me what you thought of this part of the intervention? - The aim of group exercise was to develop your confidence with exercise, increase your awareness of the benefits of exercise, help support adopting more physically active lifestyle, develop a support network, and to develop an exercise program that meets your personal needs. - What are your perceptions, do you feel that the group class achieved this or not? - If yes, tell me about your experience, if no, how does the intervention need to be changed? | - What were their thoughts on the duration, content and delivery? - The group-based session consisted of exercise and education discussions. Which elements of the group-based exercise did you find the most and least beneficial - Was there anything you felt was missing from the group exercise element of the program or is there anything you think should be removed. - Do you have any further comments that you wish to share about the group-based exercise? |
| **Self-management:** We would now like to focus on the 12 weeks of self-management you had. During this time, you had monthly contact with the fitness instructor to check up on how you were managing your exercise and physical activity.   - Can you tell me what you thought of this part of the intervention? - Overall, what impact do you think participating in PDConnect has had on you, and why? | - What were your thoughts on the duration, and contact you received during this time? - Was the monthly contact sufficient to keep you motivated to be active? |
| **Staffing: in this section we would like to focus upon the staff who delivered the PDConnect intervention. XX delivered the physiotherapy components and XX the group-based exercise and the self-management components.** | |
| - What were your thoughts of the Physiotherapist delivering the program? - What did you think of the fitness Instructor delivering the program? | - How did you find their communication, knowledge, approachability, understanding of PDConnect, supporting your involvement? |
| **The next section, we would like to explore are your perception of the study resources – this included the study manual, joint goal setting, behavior change techniques or strategies to support you to be physically active, the weekly activity planner and the activity diary.** | |
| - What were your thoughts on the PDConnect manual? During the PDConnect program the physiotherapist and fitness instructor will have set with you shared activity goals. How did you find the goal setting? - Did you use the weekly activity planner – if yes explore what they thought of it and if no – explore why not? - Did you complete the weekly activity diaries, noting your step count? – if yes explore what they thought of it and if no – explore why not? - Would you prefer to complete these diaries online or did you prefer the paper version? | - Manual- what did they think about the length, content, and level of detail? - How do you think the manual influenced your understanding of Parkinson’s? - How do you think that the manual influenced your understanding of the benefits of exercise - How do you think that the manual influenced physical activity behavior? - What were your thoughts of using goal setting, was this helpful or not? - Is there anything else you would like to comment on in relation to the study resources |
| **The next aspect we would like to explore with you is your experiences of using the Mi band activity tracker.** | |
| - What did you think of the Mi band? - Synchronization, did you experience any problems? Were the study team able to address these for you adequately? - Do you think the Mi band influenced your levels of physical activity? If yes or no, why? - Would you consider wearing it long term? | - Consider comfort ease of use, readability, functionality. |
| **Next, we are interested to hear what you thought about the RehabGuru Home exercise sheets which you received.** | |
| - Did you use RehaGuru? These were the printed instruction sheets to guide your home exercise programs. - If no, or not often why not? | - Did you find the images and instructions clear? - Were they ease to use and follow? |
| This study was originally designed to be delivered face to face. However, due to Covid it was changed to be delivered online. We are interested in your thoughts of participating in an online exercise program. | |
| - How did you find using Microsoft teams?? - Did you experience any difficulties to using Microsoft teams? If so, what were these? - Did you feel that you were able to engage with the staff delivering the intervention effectively on Microsoft Teams, if not, what could be done differently to improve this. - If you were to participate in this again, would you prefer online or face to face delivery or a combination of face to face and online, and why? | - Did you experience any challenges participating in any aspect of the PDConnect Intervention online, i.e. the 1:1 physio, the group-based exercise or the self-management aspect, if yes what were these, and how do you think these could be addressed? - In the group element of the intervention, how did you find interacting with another participants? Is there anything that could be done to enhance this experience. - Do you think anything could be changed to improve the online exercise experience? - Do you perceive any benefits with online delivery? |
| **You completed a number of questionnaires and measures at 3 different timepoints as part of this study, so we would like to next ask you about your experience in completing these measures. Some of these measures were completed on Microsoft Teams with the researcher (JJ) and the others you completed yourself online or in a paper booklet.** | |
| - The study involved a variety of measures. How did you find completing these questionnaires. - How easy was it for you to complete the questionnaires? Did you find this burdensome? - What outcome seemed the most suitable to you? - This study included a variety of measures that captured Parkinson’s symptoms, activities of daily living, physical activity and QoL. What do you think is most important for us to measure/what do you most want to see an improvement in from an intervention like this? | - What did you feel about the variety of measures that were used within the study, too much, too little? - How easy was it for you to complete the questionnaires? Did you find this burdensome? - What did you think about the frequency with which the measures were taken? - What is important to measure prompt - “is its physical activity, walking ability, general wellbeing, QoL, self-confidence, anxiety, fatigue, Parkinson’s symptoms or something else”? |
| **Other views and comments that they wish to share.** | |
| - Is there anything else that you would like to tell me about your experience of taking part in the PDConnect study? |  |

**Progression Criteria applied within the PDConnect Study Criteria**

|  | **Red** | **Amber** | **Green** |
| --- | --- | --- | --- |
| **Recruitment rate** | Less than 50% of total sample recruited in 6 months (n=<15) | Between 50% and <100% of total recruitment in 6 months (n =15- <30) | 100% of total sample (n=30) recruited in 6 months |
| **Withdrawal rate** from total sample (n=30) | More than 25% withdraw (n=8) | 15 – 25% (n=5-8) withdraw | Less than 15% (n=5) withdraw |
| **Intervention fidelity** | | | |
| **1:1 Physiotherapy n=6** | Less than 50% of 1:1 Physiotherapy sessions delivered as planned | Between 50-84% of the 1:1 Physiotherapy sessions delivered as planned | 85-100%  the 1:1 Physiotherapy sessions delivered as planned |
| **Group-based component n=12** | Less than 50% of 1:1 of the group-based classes delivered as planned | Between 50-84%  of the group-based classes delivered as planned | Between 85-100% of the group-based classes delivered as planned |
| **PDConnect Attendance rate** | | | |
| **1:1 Physiotherapy n=6** | Attends less than 50% (n≤3) of the 1:1 Physiotherapy sessions. | Attends between 50-83% (n=3-4) of the 1:1 Physiotherapy sessions. | Attends between 84-100% (n≥5) of the 1:1 Physiotherapy sessions. |
| **Group-based component n=12** | Attends less than 50% (n=6) of group sessions | Attends 50-75% (n= 4-8 of group sessions | Attends 75-100% (n≥9) of group sessions |
| **Outcome measure return rate** | | | |
| **Return of outcome measures** at 6, 18 and 30 weeks | Less than 60% of outcome measures returned at each phase | 60-80% return of outcomes measures at each phase | 80-100% of outcome measures returned at each phase |
